# Older Adults Can Suppress Unwanted Memories When Given an Appropriate Strategy

**DOI:** 10.1037/a0038611

**Published:** 2015-01-19

**Authors:** Brendan D. Murray, Michael C. Anderson, Elizabeth A. Kensinger

**Affiliations:** 1 Department of Psychology, Boston College; 2 MRC Cognition and Brain Science Unit, Cambridge, United Kingdom

**Keywords:** aging, cued recall, memory, no-think, suppression

## Abstract

Memory suppression refers to the ability to exclude distracting memories from conscious awareness, and this ability can be assessed with the think/no-think paradigm. Recent research with older adults has provided evidence suggesting both intact and deficient memory suppression. The present studies seek to understand the conditions contributing to older adults’ ability to suppress memories voluntarily. We report 2 experiments indicating that the specificity of the think/no-think task instructions contributes to older adults’ suppression success: When older adults receive open-ended instructions that require them to develop a retrieval suppression strategy on their own, they show diminished memory suppression compared with younger adults. Conversely, when older adults receive focused instructions directing them to a strategy thought to better isolate inhibitory control, they show suppression-induced forgetting similar to that exhibited by younger adults. Younger adults demonstrate memory suppression regardless of the specificity of the instructions given, suggesting that the ability to select a successful suppression strategy spontaneously may be compromised in older adults. If so, this deficit may be associated with diminished control over unwanted memories in naturalistic settings if impeded strategy development reduces the successful deployment of inhibitory control.

We often have memories we would like to forget. Memories of painful events—such as the moment we learned of the loss of a loved one—can be unwanted or unhelpful to our well-being. In other instances, information may simply be outdated and confusing if retained: The memory of where we parked last Tuesday may interfere with our ability to find our car today, for example. Regardless of the motivation driving the desire to forget, people often put intrusive memories out of conscious awareness and attempt to keep them out. Research has suggested that individuals can be quite adept at this type of controlled memory suppression and that engaging in suppression impairs later retention for the suppressed memories (Anderson & Green, 2001; Anderson & Levy, 2009; Anderson et al., 2004; Depue, Banich, & Curran, 2006; Hanslmayr, Leipold, Pastötter, & Bäuml, 2009; Murray, Muscatell, & Kensinger, 2011; Paz-Alonso, Ghetti, Matlen, Anderson, M., & Bunge, 2009; for reviews, see Anderson & Hanslmayr, 2014; Anderson & Huddleston, 2011).

*Memory suppression* refers to people’s ability to exert control over the retrieval of unwanted memories, a function often thought to be supported by inhibitory processes. The role of inhibition in suppression can be measured by studying how suppression affects the retention of traces on later tests, which is often done with the think/no-think procedure (Anderson & Green, 2001). In this paradigm, participants study pairs of semantically unrelated words, such as *ordeal* + *roach*. During study, participants are trained on these pairs until a certain criterion level of performance is achieved. Participants then engage in a think/no-think task, in which they “think” about some of those pairs while suppressing, or “not thinking,” about others. To implement this, participants are presented with trials in which they receive the left-hand word of a pair, and, while attending to the cue, they must either retrieve its paired response word (on think trials) or do all they can to keep the response word from entering consciousness (on no-think trials). Some pairs do not appear during this task and serve as baseline pairs. Following the think/no-think manipulation, participants receive an unexpected cued-recall test for all pairs. As one might expect, think pairs are recalled more often than baseline pairs on this final test. More critically, no-think pairs are recalled less often than are baseline pairs, a phenomenon known as *suppression-induced forgetting* (e.g., Anderson & Green, 2001; Anderson & Levy, 2009; Depue et al., 2006; Hanslmayr et al., 2009; Joormann, Hertel, Brozovich, & Gotlib, 2005; Lambert, Good, & Kirk, 2010; Murray et al., 2011).

Suppression-induced forgetting provides an important behavioral marker of the efficacy with which people can control the retrieval of unwanted memories. The link between this behavioral forgetting and an inhibition process is strengthened by the fact that this forgetting is cue independent (Anderson & Green, 2001). *Cue independence* refers to the tendency for the memory impairment induced by suppression to generalize to a variety of cues that one might use to access the memory trace. The presence of this cue independence is often taken as evidence of the involvement of an inhibitory control process that disrupts the memory trace itself. This property is assessed in the think/no-think procedure by giving two types of cued-recall tests: a same probe test, which uses the left-hand word from the studied pairs to cue the recall of the right-hand word of the pair (e.g., providing the cue *ordeal* for the recall of *roach*, after *ordeal* + *roach* had been studied), and an independent probe test, using an extra-list semantic cue to elicit retrieval of the response word (e.g., providing *insect* to cue *roach*). If suppression-induced forgetting generalizes to the independent probe test, this is taken as evidence that the impairment affects the suppressed item itself and not the particular association linking the study cue to the memory. Cue-independent forgetting is taken as theoretically focused evidence for the involvement of inhibitory control in suppressing the trace (Anderson & Green, 2001; for a review, see Anderson & Huddleston, 2011) and a key form of forgetting to study in the evaluation of potential inhibition deficits.

## Memory Suppression Deficits in Older Adults: Conflicting Findings

If memory suppression occurs through the use of inhibitory control, then populations with reduced inhibitory control may exhibit difficulties suppressing unwanted memories. One population believed to have impaired inhibitory control is older adults. Even in older adults without known pathology, inhibitory deficits are prevalent (for a review, see Hasher, Zacks, & May, 1999). If these deficits extended to the domain of memory suppression, this could have clinical importance (Depue, Burgess, Willcutt, Ruzic, & Banich, 2010; Joormann et al., 2005; Joormann, Hertel, LeMoult, & Gotlib, 2009; Küpper, Benoit, Dalgleish, & Anderson, 2014). Some evidence suggests that older adults may be able to control the contents of their memories despite their inhibitory deficits: Age does not always affect retrieval-induced forgetting (Aslan, Bäuml, & Pastötter, 2007; but see Ortega, Gómez-Ariza, Román, & Bajo, 2012) or listwise directed forgetting (Sego, Golding, & Gottlob, 2006; Zellner & Bäuml, 2006), despite attributions of these phenomena to an inhibitory mechanism (Anderson, Bjork, & Bjork, 1994; Anderson & Levy, 2002; Geiselman, Bjork, & Fishman, 1983; Storm & Levy, 2012). However, other evidence suggests that there may be circumstances in which older adults show impairments in memory control, such as when participants are asked to forget select items from a list rather than entire lists (Aguirre, Gómez-Ariza, Bajo, Andrés, & Mazzoni, 2014) or when attention is divided (Ortega et al., 2012). A recent study (Healey, Ngo, & Hasher, 2014) also suggested that older adults may not suppress competitors to the same extent as younger adults: When asked to generate words unrelated to a cue word (e.g., *hive*), later testing revealed that younger adults had suppressed the highly associated target word (e.g., *bee*), whereas the older adults had not.

To date, two studies have used the think/no-think procedure to compare the memory suppression ability of younger and older adults. Anderson, Reinholz, Kuhl, & Mayr (2011) investigated the effect of age on memory suppression by using the think/no-think procedure and by assessing retrieval access via same and independent probe recall tests. Because prior research had shown that age can affect circadian rhythms and the optimal time of day for cognitive processes including inhibition (e.g., Hasher, Chung, May, & Foong, 2002; Hasher et al., 1999; May & Hasher, 1998), they randomly assigned the participants to either morning or afternoon test sessions, matching the influence of this factor across groups. Although older adults did show poorer recall of no-think words than of baseline words on the same probe test, no such effect occurred on the independent probe test. Across two studies, they demonstrated that older adults did not show memory suppression when an independent probe task was used—and, in fact, older adults demonstrated numerically *better* memory for no-think words than for baseline words. As noted earlier, cue independence effects have been taken as the strongest evidence of an inhibitory suppression mechanism. Therefore, the same probe pattern was attributed to effects of interference and not considered to be evidence of inhibition. Older adults’ failure to show suppression-induced forgetting on the independent probe test is consistent with the proposed role of inhibition—and with the involvement of the dorsolateral prefrontal cortex—in memory suppression (Anderson et al., 2004; Benoit & Anderson, 2012; Benoit, Hulbert, Huddleston, & Anderson, 2015; Butler & James, 2010; Depue, Curran, & Banich, 2007; Gagnepain, Henson, & Anderson, 2014; Levy & Anderson, 2012; Paz-Alonso, Bunge, Anderson, & Ghetti, 2013). Older adults generally demonstrate a reduced ability to inhibit information and ignore distraction (Hasher & Zacks, 1988; see review by Levy & Anderson, 2008), and age-related declines in lateral prefrontal function are prevalent (Grieve, Clark, Williams, Peduto, & Gordon, 2005).

Although the results from Anderson et al. (2011) suggested that older adults are unable to suppress unwanted memories, a second study also using a think/no-think design with both same and independent probe tests revealed that older adults could succeed at memory suppression (Murray et al., 2011). In that study, on both same and independent probe tests, older adults demonstrated poorer recall of no-think words compared with both think and baseline words, and older adults’ memory suppression often did not reliably differ in magnitude from that exhibited by younger adults.

Given these conflicting findings, it is hard to determine whether older adults exhibit a deficit in suppression-induced forgetting. Understanding the conditions under which older adults can and cannot suppress information from memory may be critical to understanding the effects of age not just on memory suppression but on memory control more generally. The present study, therefore, revisited this question, considering two methodological differences between the studies of interest that may have produced the discrepant outcomes.

A first difference concerns the instructions given. When participants hear that they must put an associate out of mind on no-think trials, there are a number of strategies that they could choose to implement this instruction. Participants in Murray et al.’s (2011) study were given more explicit instructions about how to approach no-think trials than were participants in Anderson et al.’s (2011) study. In Anderson et al. (2011), participants were asked to keep the target words from coming to mind on no-think trials and were given no further information about how they might achieve this outcome. In contrast, in Murray et al. (2011), participants were given extensive examples of what *not* to do on these trials: They were asked to put the associated word out of mind and to focus on the cue word, without generating alternative associations or distracting themselves from the cue word. This methodological difference may have affected older adults’ success, because providing older adults with strategies can attenuate age-related cognitive deficits (Buckner & Logan, 2002; Naveh-Benjamin, 2000; Naveh-Benjamin, Brav, & Levy, 2007). For example, older adults have difficulty choosing effective strategies to use when encoding information (Buckner & Logan, 2002), but if they are given a strategy to use, they can implement it effectively (Buckner & Logan, 2002; Naveh-Benjamin et al., 2007). Moreover, strategy selection is likely to be a resource-demanding process, and so requiring older adults to select an effective strategy may reduce the resources available to perform the instructed task. These findings suggest that older adults’ suppression-induced forgetting deficit may be greatest when they must select the most effective strategy from among a number of alternatives or generate a strategy when none is given. If true, then providing older adults with a focused strategy should enable them to suppress unwanted memories, whereas requiring them to generate a strategy should make it difficult for them to achieve successful suppression.

A second difference between the studies is that Anderson et al. (2011) randomly assigned older and younger adults to be tested in either the morning or afternoon. In contrast, Murray et al. (2011) assigned participants to testing times based on each participant’s stated preference. The latter assignment may have led to a bias to test older adults at their optimal time of day. As discussed earlier, time of day can affect the ability to deploy inhibitory processes successfully, and so it is plausible that older adults may be able to suppress unwanted memories when tested at their optimal time of day but may struggle to do so if tested at a suboptimal time of day. Although no effect of the time of day was noted by Anderson et al. (2011), their removal of any bias toward testing older adults at their optimal time of day may have uncovered a memory suppression deficit masked in Murray and colleagues’ study by their likely assignment of older adults to an optimal testing time.

The present study sought to reconcile the conflicting findings concerning older adults’ ability to suppress unwanted memories by examining the contribution of instructions and time-of-day assignment to memory suppression ability. We elected to use the stimuli from Murray et al. (2011) to preserve consistency between that study and those presented here. For this reason, emotional and neutral word pairs were included in the present study even though word pair emotionality did not affect the pattern of results in Murray et al. and was not a key factor of interest in the present study. In Experiment 1, we used the instructions used by Anderson et al. (2011) and told younger and older adults simply to keep no-think words from coming to mind using any approach that they found effective. In Experiment 2, participants were given direct suppression instructions. These instructions emphasized that on no-think trials, they should think only about the cue word and otherwise should keep their minds blank. If the target word entered into their consciousness, they were to stop thinking about it and force it out of mind as quickly as possible, refocusing their full attention on the cue word. These instructions, modeled after those used in other studies of direct suppression (Benoit & Anderson, 2012; Bergstrom et al., 2009; Gagnepain et al., 2014; Küpper et al., 2014; van Schie, Geraerts, & Anderson, 2013), were similar to, though somewhat stricter than, those given to participants by Murray et al. (2011). In both experiments, participants were assigned to complete the study in either the morning or the afternoon (as in Anderson et al., 2011) to determine what role time of testing may play.

According to both the inhibition-deficit and strategy-deficit hypotheses, older adults should show significantly less suppression-induced forgetting compared with younger adults in Experiment 1, replicating the suppression-induced forgetting deficit reported by Anderson et al. (2011). According to the strategy-deficit hypothesis, however, this finding would not reflect a general deficit in recruiting inhibitory processes for memory suppression but, rather, a deficit in the ability to exert these processes when also required to spontaneously select a strategy, as required in Experiment 1. The two hypotheses diverge in their predictions in Experiment 2, however. According to the strategy-deficit hypothesis, when demands for strategy selection are reduced, and older adults are given an inhibition-based strategy and instructed in how to use it, they may be able to engage inhibitory control to suppress retrieval and induce suppression-induced forgetting, replicating Murray et al. (2011). In contrast, according to the inhibition-deficit hypothesis, older adults should show diminished memory suppression in Experiment 2, despite the use of more specific suppression instructions. In addition, if the time of day at which testing takes place is a factor in older adults’ memory suppression, we would expect to see suppression-induced forgetting deficits in both experiments for older adults tested at their suboptimal (afternoon) time of day.

To preview our findings, we found that younger adults showed suppression-induced forgetting whether they were given open strategy (Experiment 1) or direct suppression (Experiment 2) instructions. In contrast, when older adults were given open strategy instructions, they failed to show suppression-induced forgetting regardless of the time of day during which they are tested. However, when given direct suppression instructions and guided to a focused strategy, older adults showed successful and undiminished suppression-induced forgetting. Thus, instruction specificity matters for older but not for younger adults.

## Experiment 1

### Method

#### Participants

Participants were 22 younger adults (14 female; *M*_age_ = 19.9 years) and 44 older adults (30 female; *M*_age_ = 74.4 years) recruited from the Boston College campus and through posted advertisements in the greater Boston area. Following the methods of Anderson et al. (2011), younger and older adults were assigned randomly to be tested in either the morning (test session starting no later than 10 a.m.) or the afternoon (test session starting no earlier than 1 p.m.), without regard to their subjectively preferred time of day. Two younger adults (both p.m. group) and four older adults (two p.m., two a.m.) were discontinued because they failed to reach the learning criterion (described later). The final sample sizes were 20 younger adults (13 female; *M*_age_ = 20.0 years; nine tested in the morning and 11 tested in the afternoon) and 40 older adults (29 female; *M*_age_ = 73.9 years; 20 tested in the morning and 20 tested in the afternoon). Participants were prescreened to be in good health and have no history of depression or anxiety. Because the experimental phase contained presentation of red and green words, individuals were not run if they reported being red–green colorblind. No participants had previously performed a think/no-think task.

Additional information on the older adult participants is reported in Table 1. The older adults assigned to morning and afternoon testing times did not differ in their age, *t*(38) = 1.23, *p* = .22, Cohen’s *d* = 0.39; education, *t*(38) = 1.46, *p* = .15, Cohen’s *d* = 0.47; or time-of-day preferences, *t*(38) = 1.35, *p* = .19, Cohen’s *d* = 0.43 (as assessed via the Morningness–Eveningness Questionnaire [MEQ; Horne & Ostberg, 1976]). On the MEQ, older adults’ scores ranged from a score of 45 (neutral) to 81 (definite morning). All older adults had Mini-Mental Status Examination (MMSE) scores of 27 or higher, and these scores did not differ across participants assigned to morning and afternoon test sessions. Older adults assigned to morning and afternoon test sessions performed similarly on a large number of cognitive tasks assessing fluid and crystallized intelligence. Only the arithmetic measure (from Wechsler, 1997a) showed evidence of a group difference; older adults assigned to the afternoon test session performed better than those assigned to the morning test session, *t*(36) = 2.4, *p* = .02, Cohen’s *d* = 0.80, although this difference did not survive a Bonferroni-corrected threshold for significance.[Table-anchor tbl1]

#### Stimuli and apparatus

Materials were 160 English words, 40 negative (*M*_valence_ = 2.76 on a 1–9 scale) and 120 neutral (*M*_valence_ = 5.01 on a 1–9 scale), selected from the Affective Norms for English Words database (Bradley & Lang, 1997). Negative words were selected to be moderately arousing, with a mean arousal rating of 6.05 on a 1–9 scale. Neutral words had a mean arousal rating of 3.99. Words were randomly combined into 80 semantically unrelated word pairs, composed of a left-hand word (hereinafter referred to as a *cue* word) and a right-hand word (hereinafter, a *target* word). Cue words were always neutral, and target words could be either neutral or negative. Pairs were checked by hand to ensure that none contained semantically related referents, and the neutral cue words were varied across participants.

For the independent probe test, 80 words were chosen from the Edinburgh Word Association Thesaurus (http://www.eat.rl.ac.uk). Probe words were selected to have moderate semantic associations with one—and only one—of the 80 target words. A moderate semantic association was defined as a target word that was generated as a semantic associate for the probe word by 10%–20% of thesaurus respondents. For example, *holiday* was selected from the thesaurus as a probe word because (a) 14% of Edinburgh Word Association Thesaurus respondents generated *vacation*—a target word used in our study—as a semantic associate, and (b) *holiday* did not generate any of the other 79 target or cue words used in our study.

All stimuli were presented on a Macintosh Intel Core 2 Duo computer running MacStim 3 software (WhiteAnt Occasional Publishing, West Melbourne, Victoria, Australia). Stimuli were presented at the center of the screen in white lowercase text (48-point Lucida Grande font) on a black background.

#### Procedure

The task comprised three phases: a learning phase, a think/no-think phase with open strategy instructions, and a test phase. The test phase consisted of the same probe and independent probe tests, the order of which was counterbalanced across participants. The testing of participants on both same probe and independent probe tasks was in keeping with Anderson et al. (2011) and Murray et al. (2011) and followed the methods commonly used for the think/no-think paradigm (e.g., Anderson & Green, 2001).

##### Learning phase

The learning phase was preceded by a brief practice phase in which participants studied six pairs (comprising 12 neutral words that did not appear elsewhere in the study except during subsequent practice phases), followed by a cued-recall test for those pairs. Pairs were presented for 2,000 ms for younger adults and 5,000 ms for older adults. We used different presentation speeds to match encoding quality and overall performance levels for younger and older adults. Extensive evidence indicates age-related declines in speed of processing (e.g., Salthouse, 1996), and previous testing with this procedure has indicated that these presentation times equate depth of processing during encoding for younger and older adults (Murray et al., 2011, Experiments 3 and 4). The cued-recall test was self-paced: Participants saw single cue words appear on the screen (in a randomized order), in response to which they were asked to verbally report the corresponding target word. Responses were recorded on a laptop computer by the experimenter. Participants received both verbal and visual feedback: After they provided a response, the experimenter would tell them if they were correct or incorrect, and the correct answer would be revealed on the screen for 2,000 ms (regardless of whether the participant had responded correctly or incorrectly). Participants were told that only their first reported answer would be counted; that is, after receiving verbal feedback, they would not have the opportunity to correct their response. Incorrectly pluralized words were counted as correct (e.g., responding “lockers” if the target was *locker*).

Following practice, participants studied all 80 pairs of words, divided into four blocks of 20 pairs each. Each block consisted of a study–test cycle: Participants would study the 20 pairs and then immediately receive a cued-recall test for all 20 pairs. During each block, participants had to achieve a certain criterion level of cued-recall success to proceed to the next test block. If participants failed to reach criterion, the study–test cycle was repeated. Younger adults were held to a criterion of 50% recall performance, and older adults were held to a criterion of 65% recall performance; again, separate criteria were used for the two age groups to equate the strength of each group’s memory traces (for discussions of this rationale, see Anderson et al., 2011; Murray et al., 2011). If, within a single study block, a participant had not achieved criterion after four study–test cycles, he or she was discontinued from the study. On average, younger adults required fewer total cycles across all blocks to achieve criterion (*M* = 4.19, *SE* = 0.14) than did older adults (*M* = 5.12, *SE* = 0.33), *t*(118) = 2.59, *p* = .01, Cohen’s *d* = 0.47.

After achieving criterion on each of the four lists, participants were given a cued-recall test for all 80 of the studied pairs. Both age groups were held to a criterion of 50% on this test and were only given one opportunity to reach criterion. Participants who did not score at least 50% were discontinued. The test proceeded similarly to the previous cued-recall tests: Participants were shown a cue word, had to verbally report the corresponding target word, and were given verbal and visual feedback. Following this final test, participants were given the opportunity to take a 5-min break before continuing.

##### Think/no-think phase

During the think/no-think phase, participants were told that they would see individual cue words from the learning phase appear on the screen in either green or red. Words appeared for 4,000 ms each, separated by a 500-ms blank screen. From the original 80 cue words, 32 appeared in green text (think words), 32 appeared in red text (no-think words), and 16 did not appear at all during the experimental phase (baseline words). Although all cue words were neutral, 16 words from each color had been studied with negative targets, and 16 had been studied with neutral targets. Assignment of cue words to the think/no-think and baseline conditions was counterbalanced across participants. Each cue word was presented 10 times, for a total of 720 trials, and the order of cues was generated randomly for each individual participant. The same cue word never appeared within six presentations of itself; that is, once a word appeared, it would not be presented again until at least five other cue words had been presented. Individual cue words were always presented in the same color (i.e., think or no-think condition) for a given participant.

Participants were instructed that if a cue word appeared in green text, they were to try to think of the paired target word for the entire 4,000 ms the cue was on the screen. Even if they were sure they could not recall the associated target word, they were to try for the entire trial to call it to mind. No outward response was required, and participants were in fact instructed not to make any verbal response. They simply had to think of the associated target until the cue word disappeared. If a cue word appeared in red text, participants were given no explicit instruction for how to avoid thinking about the target word. They were told that their task was to keep the target word from coming to mind while the red cue was on the screen and that they could use whatever strategy they found to be most effective. As with the learning phase, the think/no-think phase was preceded by a brief practice: Each of the six cue words from the six practice pairs was presented (in either red or green) five times for 4,000 ms each.

After the practice session, the think/no-think phase began. During this task, every 144 trials, participants were given a brief break: The word *BREAK* would appear on the screen and remain there for 60 s, followed by a 5-s countdown during which participants were instructed to, “Get ready to continue.” For the second of these breaks, participants were given a longer break (up to 5 min) and were instructed to make a button press when they were ready to continue. After the think/no-think phase finished, participants took a 5-min break before continuing.

##### Test phase

The test phase consisted of two cued-recall tests: a same probe (SP) test and an independent probe (IP) test. The order of these tests was counterbalanced between participants. As with the cued-recall tests during the learning phase, each of these tests was self-paced: After making their verbal response, participants pressed a button to advance to the next test item. The experimenter recorded the verbal responses on a laptop computer. Unlike the cued-recall tests during the learning phase, participants were *not* given feedback as to whether they responded correctly or incorrectly.

##### SP test

The SP test proceeded identically to the cued-recall test at the end of the learning phase except that no feedback was provided. Participants were shown all 80 studied cue words, in randomized order (two randomly ordered lists were produced using a random number generator, and these test lists were counterbalanced across participants), and had to verbally provide the associated target word. Participants received a brief practice cued-recall test using the six practice pairs.

##### IP test

Participants were told that they would be tested using cue words that were not previously studied but that were semantically related in some way to target words. For example, if participants studied the practice pair *insect* + *acorn*, they would be cued with the unstudied cue *oak* + *a* . . .? In response, the participants were asked to provide the target word that was associated with the unstudied cue word (*oak*) and that started with the letter provided (*a*). Participants were given practice trials for all six practice pairs, and then cued recall was tested for all 80 targets.

##### Postexperimental survey

After the test phase, participants were given a survey asking about the frequency with which they engaged in different strategies during no-think trials. The survey, provided in the online supplemental materials, included two options that reflected the use of a retrieval suppression strategy or a strategy that stopped the retrieval process after a stimulus (the cue word) had triggered it (i.e., “[I] stared blankly at the red word and kept my mind clear” and “[I] stared intently at the red word”). Other options reflected distraction strategies that diverted attention away from the triggering stimulus (e.g., “[I] diverted my attention away from the cue word”) or that used thought substitution (e.g., “[I] used the red word to generate a personal memory”). Participants rated how frequently they used each strategy on a five-point scale from 0 (*never*) to 4 (*always*).

#### Data preparation and analysis

To ensure that differences between conditions were not contaminated by differences in the level of pair learning, we only analyzed test performance for those pairs that participants had correctly recalled during the cued-recall test at the end of the learning phase. If a participant responded incorrectly to a pair during that test, the target was excluded from analysis on the SP and IP tests (cf. Anderson et al., 2011; Murray et al., 2011).

We also sought to relate recall performance to self-reports of strategy use collected during the postexperimental survey. To investigate the effect of no-think strategy on suppression, we computed a suppression score for each participant by subtracting their cued-recall performance for no-think items from their cued-recall performance for baseline items (collapsed across emotion). This computation was performed separately for the SP and IP tests. We then took each individual’s average endorsement of the two suppression statements on the self-report survey (i.e., “[I] stared intently at the red word” and “[I] stared blankly at the red word and kept my mind clear”) and subtracted the average endorsement of all other statements; hereinafter, this is called the *selective suppression endorsement score*. The motivation for this subtraction is that the selective suppression endorsement score would be highest for participants who only, or primarily, used suppression strategies, lowest for participants who did not use suppression at all, and intermediate for participants who used suppression in conjunction with other strategies. Averages were weighted on the basis of the number of questionnaire ratings included in each computation; the suppression endorsement average was weighted by 16.67% (two statements) and the average for all others items by 83.33% (10 statements). Thus, for each participant, we had a suppression score (based on recall performance) for each of the SP and IP tests and a selective suppression endorsement score (computed from the postexperimental survey).

### Results

#### Effects of age and time of day on memory suppression

**Figure 1 fig1:**
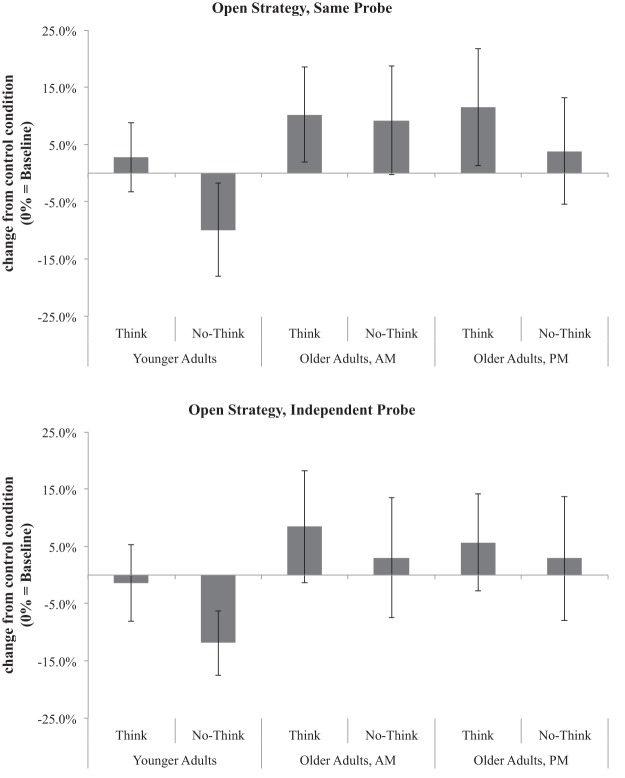
Cued-recall results for Experiment 1. Error bars represent 95% confidence intervals. Midline (0%) reflects the recall for baseline words; scores above baseline indicate cued-recall facilitation, and scores below baseline indicate cued-recall suppression. Younger adults demonstrated suppression-induced forgetting of no-think items on both the same and independent probe tests, whereas older adults showed numerical facilitation of no-think items on both tests, regardless of the time of day at which they were tested.

Data were submitted to a 3 (memory control condition: think, no-think, baseline) × 2 (target emotion: negative, neutral) × 2 (test type: SP, IP) × 3 (participant group: younger adult, morning older adult, afternoon older adult) mixed-factors analysis of variance (ANOVA). Analyses were also conducted with test order (SP first, IP first) as a factor; consistent with Anderson et al. (2011) and Murray et al. (2011), test order did not interact with any of the factors of interest and are not discussed further. The four-way interaction did not reach significance, nor did any three-way interactions (all *F*s < 1.3, all *p*s > .25, all partial η^2^s < .05). Main effects of participant group, *F*(2, 57) = 10.11, *p* < .001, partial η^2^ = .26, and memory control condition, *F*(2, 114) = 8.15, *p* < .001, partial η^2^ = .13, were observed, and these were qualified by a significant Participant Group × Memory Control Condition interaction, *F*(4, 114) = 4.09, *p* < .01, partial η^2^ = .13. As shown in Figure 1, younger adults demonstrated significant below-baseline recall for no-think words, *t*(19) = 3.81, *p* = .001, Cohen’s *d* = 0.78, whereas older adults did not, regardless of whether they were tested in the morning (when they showed numerical *facilitation* for no-think words; *t*[19] = 1.67, *p* = .11, Cohen’s *d* = 0.38) or in the afternoon, *t*(19) = 0.83, *p* > .40, Cohen’s *d* = 0.16, replicating Anderson et al. (2011). No other main effects or interactions achieved significance (all *F*s < 2.1, all *p*s > .12, all partial η^2^s < .07). Means for all three participant groups, separated by test type, are reported in Table 2.[Fig-anchor fig1][Table-anchor tbl2]

#### Relationship between memory suppression performance and self-reported use of suppression strategies

As described in the Method section, participants were asked to report what type of no-think strategies they used, and a selective suppression endorsement score was derived. A higher score indicated that a participant was more likely to spontaneously use a suppression strategy (i.e., “[I] stared blankly at the red word and kept my mind clear” and “[I] stared intently at the red word”), and a lower score indicated that the participant was more likely to use a distraction (e.g., “[I] diverted my attention away from the cue word”) or substitution strategy (e.g., “[I] used the red word to generate a personal memory”). On average, older adults were less likely to use suppression compared with other strategies, as reflected by an overall negative selective suppression endorsement score (*M*_SSE_ = −0.54, *SD* = 0.61). There was no difference in selective suppression endorsement scores between older adults tested in the morning (*M*_strategy_ = −0.68, *SD* = 0.63) or afternoon (*M*_strategy_ = −0.40, *SD* = 0.57), *t*(38) = 1.43, *p* > .15, Cohen’s *d* = 0.46.

**Figure 2 fig2:**
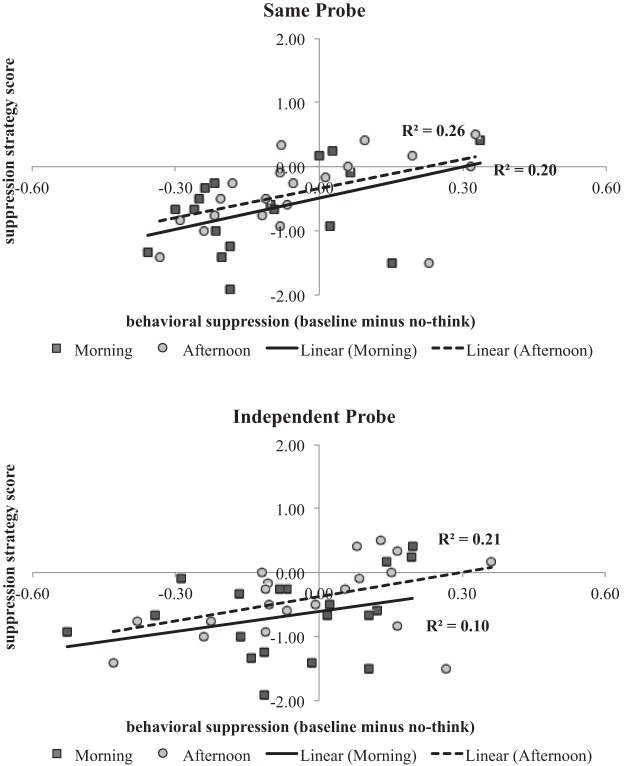
Relationship between self-reported suppression strategy and behavioral suppression score for older adults tested in the morning and in the afternoon. On both same probe and independent probe tasks, a significant relationship was observed between selective suppression endorsement and behavioral suppression: An increase in selective endorsement of suppression strategies was related to better behavioral suppression (i.e., baseline minus no-think).

Separate linear regression models were run with selective suppression endorsement score as the predictor variable and either SP suppression score (i.e., baseline minus no-think) or IP suppression score as the dependent variable. For both tests, selective suppression endorsement significantly predicted behavioral memory suppression: Higher suppression strategy scores were related to better behavioral suppression on the SP (β = 0.50), *t*(38) = 3.56, *p* = .001, *r*^2^ = .25, and IP tests (β = 0.40), *t*(38) = 2.66, *p* = .01, *r*^2^ = .16. The older adult data for the SP and IP tests are displayed in Figure 2.[Fig-anchor fig2]

Younger adults, in comparison, demonstrated no relationship between selective suppression endorsement and suppression scores (i.e., baseline minus no-think) on either SP, *t*(19) = 0.67, *p* > .50, *r*^2^ = .02, or IP tests, *t*(19) = 0.23, *p* > .80, *r*^2^ = .003. However, younger adults endorsed suppression strategies to a significantly greater degree than did older adults, *t*(58) = 5.00, *p* < .001, Cohen’s *d* = 1.31, and *t*(58) = 5.71, *p* < .001, Cohen’s *d* = 1.49, for the two suppression questions, respectively. Moreover, of the 20 younger adults tested, all reported at least some use of a suppression strategy. The lack of a correlation, therefore, was likely driven by the fact that *all* younger adults reported using a suppression strategy to some degree. Younger adults’ variability in task performance may have had less to do with whether they recruited suppression strategies and more to do with how effectively they used them.

It is worth noting that, overall, younger adults’ sum total strategy endorsement (when endorsement for all items is added together: *M* = 14.45, *SD* = 3.7) was higher than older adults’ total endorsement (*M* = 10.38, *SD* = 5.1). This could suggest that younger adults may be more biased toward endorsing strategies than older adults, or—more interestingly—that younger adults may access a broader range of strategies to try and suppress effectively, whereas older adults may sometimes be unable to select a strategy. The average endorsement for each survey item can be seen in Table 3.[Table-anchor tbl3]

#### Median split analysis

One potential concern may be that older adults are less homogenous than younger adults in their overall memory performance. In the think/no-think task, participants must be able to remember a large number of pairs from the initial learning phase to the test phase, and suppression ability may be influenced by how many baseline items participants are able to hold in mind over that time period. Indeed, we observed much variability in memory performance for baseline items among older adults: On the SP and IP tests, older participants remembered anywhere from 0% to over 90% of the baseline items that they had successfully remembered during the learning phase (compared with a range of 50%–94% for younger adults). Older adults who are better at retaining information over a delay may also be better at cognitive control tasks, and so one could imagine a case in which a few “high-memory” older adults demonstrated suppression, whereas “low-memory” older adults did not.

To explore this possibility, we took the older adults with the six highest and six lowest baseline memory scores on the IP and SP tests from both the morning and afternoon groups and looked to see if they differed in their suppression ability on either test. The mean no-think and baseline scores can be seen in Table 4. Whereas the high-memory older adults did have numerically higher baseline scores than the low-memory older adults (although, because of a lack of power, these differences did not achieve significance), the high-memory older adults still showed no suppression of no-think items.[Table-anchor tbl4]

### Discussion

Replicating the findings of Anderson et al. (2011), older adults were significantly impaired in their ability to suppress unwanted memories, compared with younger adults, under unguided conditions in which participants had to self-generate suppression strategies. In fact, although the no-think condition did not differ significantly from baseline pair recall, recall of no-think words was numerically *higher* than for baseline items, as it was in Anderson et al. (2011). This was true regardless of the time of day at which older adults were tested. In fact, the evidence for an age-related memory suppression deficit in Experiment 1 is more general than that reported by Anderson et al. (2011): Whereas they observed an inhibition deficit only on the IP test, here we observed deficits when using either SP or IP tests. We also observed equivalent inhibition deficits for emotional and neutral pairs (see the online supplementary material for separate analyses with emotional and neutral pairs). The failure of this inhibition deficit to be moderated by time of day or emotion suggests that the differences between Anderson et al., (2011) and Murray et al. (2011) on these dimensions may not have been the critical variables explaining their discrepant findings.

Consistent with previously reported findings (Anderson & Green, 2001; Anderson & Levy, 2009; Depue et al., 2006; Hanslmayr et al., 2009; Joormann et al., 2005; Lambert et al., 2010; Murray et al., 2011), younger adults demonstrated significant suppression-induced forgetting of no-think words. This occurred despite the fact that younger adults were given the same open-ended instructions as were older adults. As noted in the Results section and shown in Table 3, younger adults endorsed suppression survey items significantly more often than did older adults. These results suggest that even when younger adults are given open-ended suppression instructions, they are more likely than older adults to spontaneously recruit suppression strategies. Importantly, older adults who endorsed spontaneous use of suppression strategies (e.g., “[I] stared blankly at the red word and kept my mind clear”) were more likely to show a memory suppression effect than those older adults who did not endorse such strategies.

These findings support the idea that the key factor explaining the discrepant findings of Anderson et al. (2011) and Murray et al. (2011) concerns the spontaneous use of suppression strategies by older adults. However, retrospective reports of strategy use may suffer from inaccuracies, and, thus, the validity of this explanation required further testing. If older adults’ failure to suppress in Experiment 1 reflected a failure to spontaneously adopt a successful suppression strategy, then if older adults are given more explicit instructions on how to suppress information, they should show a memory suppression effect. Experiment 2 was conducted to test this hypothesis. Participants were given explicit instructions during the think/no-think phase for how to suppress no-think words. Following on prior work (Benoit & Anderson, 2012; Bergström, de Fockert, & Richardson-Klavehn, 2009), participants were asked to (a) focus on the red word on the screen, (b) keep its paired associate from coming to mind, (c) force the associate out of mind immediately if it did spontaneously come into consciousness, and (d) not generate substitute thoughts. To preview our findings, under these more directed instructions, all three groups—younger adults, older adults tested in the morning, and older adults tested in the afternoon—demonstrated significant suppression-induced forgetting of no-think items.

## Experiment 2

### Method

#### Participants

Participants were 20 younger adults (11 female; *M*_age_ = 20.0 years) and 48 older adults (29 female; *M*_age_ = 74.7 years) recruited from the Boston College campus and through posted advertisements in the greater Boston area. No participants had previously performed a think/no-think task. Whenever possible, participants were randomly assigned to be tested in either the morning or the afternoon. Because of recurring scheduling conflicts, 12 older adults were only available to participate during one timeslot (eight in the morning and four in the afternoon) and were scheduled accordingly rather than being excluded from the study. Eight older adults (seven p.m., one a.m.) were discontinued because they failed to reach the learning criterion, for a final sample size of 40 older adults (27 female; *M*_age_ = 74.3 years; 20 tested in the morning and 20 tested in the afternoon). Twelve younger adults were tested in the morning, and eight were tested in the afternoon. As in Experiment 1, all participants were prescreened to be in good health, have no history of depression or anxiety, and have no red–green colorblindness.

The older adult participants enrolled in Experiment 2 completed the same questionnaires and cognitive tests as those enrolled in Experiment 1 (see Table 1). The participants enrolled in Experiment 2 did not differ from the participants enrolled in Experiment 1 on any of these measures. As in Experiment 1, all older adult participants enrolled in Experiment 2 had MMSE (Folstein, Folstein, & McHugh, 1975) scores of 27 or higher. They all endorsed being “neutral” to “definite morning” types on the MEQ (Horne & Ostberg, 1976), and there was no difference in the MEQ scores between older adults tested in the morning and those tested in the afternoon (*t* < 1). The older adults tested in the morning and afternoon performed similarly on a large number of cognitive tasks assessing fluid and crystallized intelligence; there were no significant differences in these measures between participants tested in the morning and in the afternoon test sessions (all *t*s < 1.4, all *p*s > .15, all Cohen’s *d*s < 0.42).

#### Stimuli and procedure: Direct suppression instructions

The stimuli and procedure were similar to those in Experiment 1, with a few exceptions. First, and most important, the instructions given during the experimental phase were different for Experiment 2 than for Experiment 1. For the think/no-think phase, participants were given explicit instructions for how to suppress target words associated with red cue words. Participants were told that when they saw a red cue word, they were to clear their mind entirely of the associated target word and focus their full attention on the cue word for the entire time it was on the screen. They were instructed *not* to think about other potential associates for the cue word, to play word games with the cue word, to repeat the cue word over and over in their mind, to shift their eyes away from the word or from the screen, or to do anything else that would distract them from thinking about the cue word. It was emphasized that they should think only about the cue word and otherwise keep their minds blank. If the target word entered into consciousness, they were to stop thinking about it, force it out of mind as quickly as possible, and refocus their full attention on the cue word.

A brief practice phase, using filler word pairs, was given to ensure that participants could use the instructions given. Following the practice phase, participants were given a brief diagnostic questionnaire about what strategies they were using during the no-think practice trials. They were asked how frequently (on a five-point scale, with 1 being *never* and 5 being *always*) they did things like keep their mind totally clear and focus on the cue word, think distracting thoughts to prevent the target from coming to mind, look away from the screen, and so on. When participants reported engaging in any of the alternative strategies (i.e., any strategy other than focusing on the cue word), they were reminded of the task instructions. This same questionnaire was also administered during the long break during the think/no-think phase. Finally, following the final test phase at the end of the experiment, participants were given a brief compliance questionnaire to determine whether they had intentionally disregarded the no-think instructions (e.g., “When I saw a red cue word, I thought about the target word that went with it *in an effort to improve my memory for that pair*”). There were three such questions that participants were asked to respond to on a four-point scale from 0 (*never*) to 3 (*frequently*), and we set an a priori criterion for exclusion if a participant scored higher than a total of 5 across all three questions. No participant scored high enough for exclusion, and most scored a total of 0. This compliance questionnaire replaced the postexperimental strategy questionnaire (see Table 3) given in Experiment 1; we eliminated the strategy questionnaire because we believed participants’ responses would be too heavily influenced by demand characteristics to be valid because participants were given explicit instructions on what strategy to use and on which to avoid.

The new diagnostic questionnaire given during the practice phase of the think/no-think task was designed to ensure that participants were following the specialized direct suppression instructions and were not deviating from the prescribed strategy. We therefore assessed their success after a practice set of think/no-think trials that preceded the start of the full think/no-think phase. In Experiment 1, because participants were free to engage with the no-think words in whatever way they found to be effective, there was no strategy compliance to diagnose; further, we did not want to give participants any ideas for potential strategies that they could use so as to leave strategy selection purely up to them.

Aside from these changes, Experiment 2 proceeded identically to Experiment 1. Recall data were conditionalized in the same manner described for Experiment 1.

### Results

#### Effects of age and time of day

Data (summarized in Table 5) were submitted to a 3 (memory control condition: think, no-think, baseline) × 2 (target emotion: negative, neutral) × 2 (test type: SP, IP) × 3 (participant group: younger adult, morning older adult, afternoon older adult) mixed-factors ANOVA. Analyses were also conducted with test order (SP first, IP first) as a factor; test order did not interact with any of the factors of interest and is not discussed further. Once again, the four-way interaction did not reach significance, nor did any three-way interactions (all *F*s < 1.4, all *p*s > .25, all partial η^2^s < .06). Main effects were observed for memory control condition, *F*(2, 114) = 60.12, *p* < .001, partial η^2^ = .51; test type, *F*(1, 57) = 87.14, *p* < .001, partial η^2^ = .61; and participant group, *F*(2, 57) = 4.45, *p* = .02, partial η^2^ = .14. No-think words were recalled at a significantly lower rate than were baseline words (SP: *t*[59] = 6.98, *p* < .001, Cohen’s *d* = 1.81; IP: *t*[59] = 5.24, *p* < .001, Cohen’s *d* = 1.36) and think words (SP: *t*[59] = 10.00, *p* < .001, Cohen’s *d* = 2.60; IP: *t*[59] = 7.94, *p* < .001, Cohen’s *d* = 2.06). More SP words (*M* = 77.1%, *SE* = 1.4%) were recalled than IP words (*M* = 56.6%, *SE* = 1.5%), and older adults tested in the afternoon recalled fewer items than younger adults, *t*(38) = 2.74, *p* < .01, Cohen’s *d* = 0.89, and older adults tested in the morning, *t*(38) = 2.08, *p* = .05, Cohen’s *d* = 0.67.[Table-anchor tbl5]

**Figure 3 fig3:**
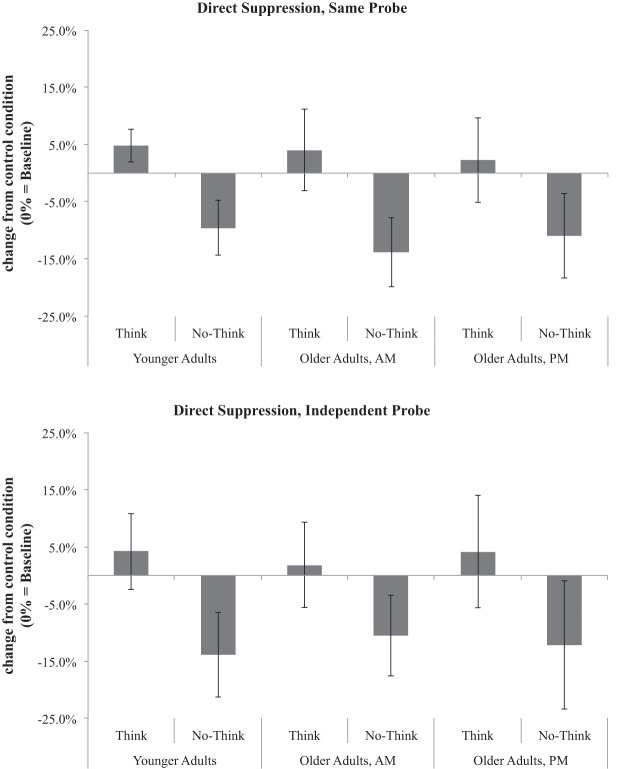
Cued-recall results for Experiment 2. Error bars represent 95% confidence intervals. Midline (0%) is the recall for baseline items; scores above baseline indicate cued-recall facilitation, and scores below baseline indicate cued-recall suppression. Younger and older adults both demonstrated significant suppression of no-think items on both tests.

Critically, participant group did not interact with any of the variables of interest. Shown in Figure 3, all three groups showed no-think recall performance that was significantly below baseline. This was true for both the SP and IP tests, regardless of the emotionality of the word being suppressed (negative or neutral). Thus, these findings replicate Murray et al. (2011) in showing intact memory suppression in older adults, and they contrast with the results of Experiment 1.[Fig-anchor fig3]

#### Comparison of Experiment 1 and Experiment 2

Although Experiments 1 and 2 were run separately from one another, it is informative to compare them. The participants in the two experiments were recruited in the same manner, and the older adult participants enrolled in the two studies did not differ on demographic or cognitive test scores (see Table 1). Given the visibly distinct results found for older adults under the two instruction conditions, it was important to examine whether the apparent interaction of memory suppression ability with instruction condition achieved statistical significance.

##### Effects of instruction manipulation, age, and time of day

Data were submitted to a 3 (memory control condition: think, no-think, baseline) × 2 (target emotion: negative, neutral) × 2 (test type: SP, IP) × 3 (participant group: younger adult, morning older adult, afternoon older adult) × 2 (instruction: open strategy, direct suppression) mixed-factors ANOVA. The five-way interaction did not reach significance, nor did any four-way interactions (all *F*s < 1.6, all *p*s > .15, all partial η^2^s < .04). However, a significant three-way interaction was observed between memory control condition, participant group, and instruction, *F*(4, 228) = 2.63, *p* = .04, partial η^2^ = .04. Whereas younger adults showed significant suppression (no-think < baseline) in both the open strategy and direct suppression instruction conditions, older adults demonstrated suppression only in the direct suppression condition. This was true regardless of whether older adults were tested in the morning or the afternoon. To unpack this interaction, we submitted each participant group to separate 3 (memory control condition) × 2 (target emotion) × 2 (test type) × 2 (instruction) mixed-factors ANOVAs. Note that in the following paragraphs, we use the nomenclature of “*x* = *y* > *z*”, for example, to indicate that the subsequent paired *t* test of the simple effect is significant at a Bonferroni-corrected alpha level of *p* < .05. In this example, conditions *x* and *y* would not significantly differ, but *x* and *y* would each be significantly greater than *z*.

##### Older adults, a.m. testing

Older adults tested in the morning demonstrated main effects of instruction (open strategy vs. direct suppression), *F*(1, 38) = 8.61, *p* < .01, partial η^2^ = .19; memory control condition, (think > baseline = no-think), *F*(2, 76) = 9.93, *p* < .001, partial η^2^ = .21; and test type (SP > IP), *F*(1, 38) = 8.49, *p* < .01, partial η^2^ = .18. Critically, however, memory control condition interacted with instruction, *F*(2, 76) = 9.81, *p* < .001, partial η^2^ = .21. Older adults who received direct suppression instructions showed a significant suppression effect (think = baseline > no-think), *F*(2, 38) = 21.43, *p* < .001, partial η^2^ = .53, whereas those who received open strategy instructions actually showed numerical *facilitation* for no-think items over baseline items, *t*(19) = 1.67, *p* = .11, Cohen’s *d* = 0.38. For both the direct suppression and open strategy instruction groups, test type did not interact with these effects (all *F*s < 1), indicating that this pattern held across both the SP and IP tests.

##### Older adults, p.m. testing

Older adults tested in the afternoon demonstrated main effects of memory control condition (think > baseline > no-think), *F*(2, 76) = 9.49, *p* < .001, partial η^2^ = .20, and test type, (SP > IP), *F*(1, 38) = 23.56, *p* < .001, partial η^2^ = .38). Although there was no main effect of instruction (*F* < 1), instruction interacted significantly with memory control condition, *F*(2, 76) = 5.40, *p* < .01, partial η^2^ = .12). Here again, older adults who received direct suppression instructions showed significant suppression (think = baseline > no-think), *F*(2, 38) = 12.85, *p* < .001, partial η^2^ = .40, whereas those who received open instructions did not (think > no-think = baseline), *F*(2, 76) = 3.18, *p* = .05, partial η^2^ = .14. Again, test type did not interact with any factors (all *F*s < 1). No other interactions reached significance for afternoon older adults (all *F*s < 2, all *p*s > .15, all partial η^2^s < .05).

##### Younger adults

Younger adults demonstrated significant main effects of memory control condition (think = baseline > no-think), *F*(2, 76) = 38.76, *p* < .001, partial η^2^ = .51, and test type (SP > IP), *F*(1, 38) = 35.63, *p* < .001, partial η^2^ = .48. Younger adults demonstrated no main effect of instruction, *F*(1, 38) = 2.50, *p* = .12, partial η^2^ = .06, and, critically, instruction did not interact with memory control condition, *F*(2, 76) = 1.08, *p* = .34, partial η^2^ = .028, indicating that the main effect of memory control condition held regardless of instruction condition. Instruction interacted significantly with test type, *F*(1, 38) = 22.42, *p* < .001, partial η^2^ = .37, but no other factors: Younger adults who received direct suppression instructions performed better on the SP test than the IP test, whereas those who received open strategy instructions performed equivalently on both tests.

### Discussion

In contrast to Experiment 1, older adults in Experiment 2 demonstrated suppression-induced forgetting of no-think items. Although overall main effects of test type and participant group were observed, older adults did not differ from younger adults in their memory suppression for either emotional or neutral items (see the online supplementary materials for separate reporting for emotional and neutral items). Older adults who were tested in the afternoon did recall fewer items overall than either younger adults or older adults tested in the morning, consistent with prior evidence that afternoon tends to be a suboptimal time of day for older adults’ cognitive performance (Yoon, May, & Hasher, 1999). Nevertheless, it is unexpected that the *amount* of information that older adults can retain in memory is adversely affected by afternoon testing but *not* older adults’ ability to implement memory suppression strategies believed to rely on inhibitory control ability.

Indeed, in the present studies, circadian effects did not appear to affect the ability to suppress already-learned information. Older adults tested in the afternoon were able to suppress no-think items at a rate that did not differ from that of their morning counterparts or younger adults. This result informs several domains of cognitive research: First, as has previously been argued (Hasher et al., 1999; May & Hasher, 1998) time-of-day effects do not necessarily extend indiscriminately to all tasks. In this case, time of testing related to older adults’ overall recall rates but not their ability to exercise cognitive control over that information. Second, as elaborated in the General Discussion section, this result suggests that previously reported inhibitory deficits for older adults (Hasher & Zacks, 1988) can be overcome under certain circumstances, such as giving older adults the support of direct suppression instructions as in the present task.

There are two potential limitations that warrant mention. The first is that a subset of the older adults were scheduled for the only testing time (morning or evening) during which they were available; this prevented a fully randomized assignment, as had been achieved in Experiment 1. Although there is no evidence from MEQ scores or task performance that the older adults who chose afternoon testing times were doing so because it was their preferred time of day, it is possible that this nonrandom assignment minimized time-of-testing effects. A second potential limitation relates to the comparison of the Experiment 1 and Experiment 2 data. As in any between-subjects design, it is possible that the effect of instruction (open strategy vs. direct suppression) resulted not from the instruction manipulation but from other differences between the groups of older adults assigned to the two instruction conditions. We think this alternate explanation is unlikely for two reasons. First, the cognitive test scores of the older adult participants (see Table 1) provide no evidence of differences in the participants who were enrolled in the two experiments. Second, within a single group of participants in Experiment 1, there was a correlation between suppression score and endorsed strategy but no correlation between suppression score and either baseline-item performance or participant age. This pattern suggests that it is likely the instructed strategy that created the performance differences between Experiment 1 and Experiment 2 rather than other demographic or cognitive factors.

## General Discussion

The diverging patterns of suppression-induced forgetting across Experiments 1 and 2 suggest that older adults’ tendency to engage the inhibitory control processes necessary for effective memory suppression depends on the instructional support offered. In Experiment 1, participants received open strategy instructions, in which they were told to suppress awareness of no-think words by whatever means they found viable. Under these conditions, which provided little instructional direction, older adults had difficulty suppressing no-think words and, in fact, often showed *above*-baseline remembering for to-be-suppressed items. Facilitation of no-think items may be attributable to a compounding effect of failed suppression: If the to-be-suppressed item comes to mind and is not inhibited, the item may actually be *reinforced* through its re-encoding. The self-report survey after the experiment suggested that suppression may have failed, in part, as a result of older adults’ tendency to rely on self-distraction rather than strategies that might engage inhibitory control. Those older adults who did report spontaneously using strategies in line with direct suppression demonstrated the most suppression success. This interpretation of the memory suppression deficit observed in Experiment 1 is supported by the findings of Experiment 2: When participants were given specific direct suppression instructions—along with corrective feedback before and during the think/no-think phase—both older and younger adults showed significant suppression effects that did not vary reliably in magnitude.

The finding that instructional support moderates whether older adults exhibit a deficit in memory suppression helps to reconcile the discrepant findings of Anderson et al. (2011) and Murray et al. (2011). When we used Murray et al.’s same materials but Anderson et al.’s (2011) open-ended no-think instructions, we fully replicated the evidence for an age deficit in suppression-induced forgetting that Anderson et al. (2011) observed. Indeed, the age deficit in memory suppression observed in Experiment 1 was more general than that observed by Anderson et al. (2011), extending across both the SP and IP tests and occurring for emotional and neutral information (see the online supplemental materials for reporting of results for emotional and neutral pairs). In contrast, using the same materials but instructions that asked participants to use a direct suppression strategy, we replicated Murray et al.’s finding that older adults exhibit intact suppression-induced forgetting for emotional and neutral information. Although, in the Murray et al. study, participants were not given direct suppression instructions per se, they may have been pushed in that direction: Participants were instructed *not* to engage in many of the behaviors that older adults endorsed in Experiment 1 of the present study, such as distraction or substituting the to-be-suppressed word for a different word. Providing older adults with strategies for how to complete cognitive tasks can often attenuate age-related cognitive deficits (Buckner & Logan, 2002; Naveh-Benjamin, 2000; Naveh-Benjamin et al., 2007). Providing detailed instructions for how to suppress information may be another instance of this finding.

Although differences in time of testing had seemed to be another potential explanation for the differences between Anderson et al. (2011) and Murray et al. (2011), we found no evidence that this factor influenced older adults’ success at memory suppression. Time of testing did affect older adults’ ability to learn the word pairs (Experiments 1 and 2) and to reach criterion (Experiment 2): Older adults tested in the afternoon had more difficulty learning the pairs than did older adults tested in the morning. Yet in neither experiment did time of testing influence older adults’ memory suppression ability. Regardless of the time of testing, older adults in Experiment 1 failed to show memory suppression, and older adults in Experiment 2 showed intact memory suppression ability. Thus, whether an inhibition deficit is observed (as in Experiment 1) or not (as in Experiment 2) appears to hinge on whether older adults are given a direct suppression strategy to use and not on the time of day at which they are tested.

These findings clarify and extend the research of Murray et al. (2011) and Anderson et al. (2011). In contrast to the conclusions of Murray et al., the present results reveal that older adults do indeed have difficulties with memory suppression. When given no specific instructions for how to suppress, they fail to suppress unwanted memories. Yet the present results also suggest that these difficulties may arise not because of older adults’ inability to execute the inhibition processes needed for successful suppression, as originally suggested by Anderson et al. (2011), but rather because older adults fail to spontaneously adopt a memory suppression strategy that engages those processes. Thus, it may not be that older adults *cannot* inhibit unwanted memories but, rather, that they *tend not to* because they have difficulties deploying this strategy spontaneously.

These results suggest that, in daily life and when no external support is available to direct older adults toward an appropriate strategy, older adults are likely to exhibit a memory control deficit. It will be important for future research to examine the implications of this deficit for older adults’ daily functioning. Failures in memory suppression are correlated with symptom severity in attention-deficit/hyperactivity disorder (Depue et al., 2010) and are prevalent in individuals with depression, particularly when the unwanted memories are of negative valence (LeMoult, Hertel, & Joormann, 2010). Thus, older adults’ failure to engage the appropriate strategies for memory suppression may have clinical relevance.

A question raised by the present study is why the strategies that older adults spontaneously engage do not seem to produce successful memory suppression. Older adults endorsed using strategies, such as self-distraction, which have previously been shown to induce forgetting (Sahakyan, Delaney, & Goodmon, 2008). One possibility is that age-related slowing leads older adults to take longer to implement any particular strategy. Even though they endorse a strategy such as self-distraction, they may not engage it quickly enough on a trial-by-trial basis for it to prevent the unwanted information from coming to mind, or they may have insufficient time to deploy the strategy effectively. In this context, it is worth highlighting the fact that in the present study, younger and older adults were given the same amount of time on each trial in the think/no-think portion of the task. We equated trial duration in this manner because otherwise the delay length, or the portion of the delay filled by the think/no-think task, would necessarily differ between younger and older adults, which could create its own confounds. Yet it would be interesting for future research to assess whether older adults can suppress memories successfully—even when no strategy is instructed—if the trial lengths during the think/no-think phase are elongated, perhaps giving them sufficient time to deploy their selected strategies. It is also possible that older adults are less likely than are younger adults to think that they need to come up with any systematic strategy to suppress unwanted memories. It has previously been shown that in a listwise directed forgetting paradigm, older adults were less likely than younger adults to develop a strategy to help them forget information because they felt they did not need to do anything special to forget (Sahakyan et al., 2008). A similar age difference could explain the present results. Indeed, although older adults did endorse using strategies (see Table 3), their rates of endorsement generally were low—lower than those of younger adults. Thus, the failure of older adults to suppress unwanted memories may have reflected their tendency to think that forgetting would likely occur even in the absence of strategy engagement.

It should be noted that although the present results suggest that a strategy-use failure, rather than a pure inhibitory deficit, is the likely explanation for older adults’ deficits in memory suppression, these results cannot rule out the possibility that there is a subset of older adults, or a set of circumstances, in which failure to suppress arises from a pure inhibitory deficit. For instance, when older adults are divided into those over (“oldest-old”) or under (“youngest-old”) the age of 75 or 80, the “oldest-old” show inhibition deficits that go beyond those present in the youngest-old (Aslan & Bäuml, 2012, 2013). It is possible that the oldest-old—at the upper limits of the age range tested here—would show deficits in suppression-induced forgetting because of their inability to use inhibitory mechanisms even when instructed. It is also possible that even the youngest-old would be unable to execute inhibitory processes if asked to suppress unwanted information while their attentional resources were taxed (for related evidence in retrieval-induced forgetting, see Ortega et al., 2012; Román, Soriano, Gómez-Ariza, & Bajo, 2009). In fact, one alternate interpretation of the results of the present study is that, by requiring participants to select their own strategy, Experiment 1 placed a high demand on participants’ attentional resources. Under this attentional demand, older adults may not have had the resources to implement the inhibitory processes needed for memory suppression. By removing the burden of selecting a strategy, Experiment 2 may have reduced the attentional demands of the task, making more resources available for execution of the inhibitory processes that support memory suppression. This explanation would be consistent with cognitive and neural theories of aging (e.g., Fabiani, 2012; Reuter-Lorenz & Cappell, 2008) that emphasize the exaggeration of age-related deficits when resource demands are high.

Although the present study has demonstrated that older adults can harness an inhibitory strategy to suppress memories, these results should not be taken as evidence that older adults demonstrate no inhibition deficits. Instead, what these results suggest is that under certain conditions—such as when older adults are directed to an inhibition-focused strategy, thereby reducing strategy-selection demands—their deficits are not great enough to impair their ability to implement inhibitory processes to achieve successful memory suppression. As described earlier, older adults may succeed at memory suppression in a directed suppression condition but not an open strategy condition either because the directed instructions correct an age-related deficit in selecting an effective strategy or because, by eliminating the need for strategy selection, the instructions reduce the cognitive demands of the task and thus enable older adults to devote those resources toward inhibitory processes. This latter suggestion is generally consistent with the findings of Aguirre et al. (2014), who demonstrated that older adults showed an impaired ability to voluntarily forget episodic memories when the task required selective forgetting but performed as well as younger adults when the task required listwise forgetting. It also is consistent with recent experiments by Ortega et al. (2012), who showed that younger and older adults show equivalent retrieval-induced forgetting under conditions of full attention but show evidence of inhibitory impairments under conditions of divided attention. These studies have been interpreted as providing evidence that age differences in cognitive control are easier to detect when tasks have high requirements for executive control (Aguirre et al., 2014), a proposal that is consistent with both cognitive (e.g., Craik, 1986) and neural (e.g., Fabiani, 2012; Reuter-Lorenz & Cappell, 2008) theories of aging.

Our argument that people can adopt an inhibitory strategy should not be taken to suggest that inhibition is not a fundamental mechanism of cognitive control necessary in a range of cognitive tasks. A distinctive feature of voluntary retrieval suppression, as studied in the think/no-think task, is that it overtly emphasizes excluding a memory from awareness, often without detailing how this should be achieved. This is unlike many other cognitive control tasks in which participants perform a specific task (i.e., retrieving a target memory), and regulation of competing memories is an incidental demand of task performance. Without specific strategy instructions, the no-think task requires the participants to specify the nature of the mental activities they will engage in to exclude a memory from awareness. These activities may vary in the extent to which they require inhibitory control to be implemented well. More specific instructions, like direct suppression, weight inhibitory control more heavily than others. In other tasks used to study inhibitory control (e.g., retrieval practice), there is less latitude in how a task is performed, and the demands placed on inhibition mechanisms may be more consistent. It remains unexplored, however, whether even in relatively more constrained tasks, the engagement of inhibition may be modulated voluntarily.

In conclusion, the current experiments present strong evidence that older adults’ deficits in memory suppression arise not from an inability to implement inhibitory strategies but, rather, from a failure to select such strategies. With strategic support, older adults can inhibit memories as effectively as do younger adults. These findings help to reconcile findings in the literature that, to this point, have appeared inconsistent as to whether older adults can suppress learned information. This research further informs theories of how advancing age affects memory, demonstrating that older adults’ difficulties controlling the contents of their memories can be caused by failures in strategy selection rather than by a pure inhibitory deficit.

## Supplementary Material

10.1037/a0038611.supp

## Figures and Tables

**Table 1 tbl1:** Characteristics of Participants (Means, With Standard Deviations in Parentheses) in Experiments 1 and 2

	Experiment 1		Time-of-day comparison		Experiment 2		Time-of-day comparison	
Variable	a.m.	p.m.	*t*	*p*	a.m.	p.m.	*t*	*p*
Age (years)	75.3 (7.8)	72.5 (6.4)	*t*(38) = 1.24	.22	73.7 (7.2)	75.0 (6.2)	*t*(38) = 0.57	.57
Education (years)	15.9 (2.3)	16.9 (2.4)	*t*(38) = 1.46	.15	15.7 (2.2)	15.7 (2.2)	*t*(38) = 0.07	.94
MEQ (max. = 86)	64.3 (8.4)	60.6 (8.7)	*t*(38) = 1.3	.19	59.9 (8.9)	58.6 (9.0)	*t*(38) = 0.48	.64
MMSE (max. = 30)	29.2 (0.8)	29.0 (0.8)	*t*(38) = 0.39	.70	29.0 (1.0)	29.0 (0.9)	*t*(38) = 0.0	1.0
Shipley vocabulary (max. = 40)	35.5 (3.7)	34.9 (4.8)	*t*(38) = 0.37	.71	37.2 (3.2)	35.8 (3.0)	*t*(38) = 1.4	.17
Arithmetic (Max. = 22)	14.0 (3.8)	16.7 (3.0)	*t*(36) = 2.4	.02	14.8 (2.6)	14.8 (3.6)	*t*(37) = 0.09	.93
Digit symbol (max. = 93)	28.7 (11.0)	29.2 (11.7)	*t*(38) = 0.13	.90	33.0 (6.0)	34.6 (7.6)	*t*(38) = 0.74	.47
Digit span backwards (max. = 14)	7.6 (1.9)	7.8 (2.5)	*t*(37) = 0.34	.74	8.9 (2.4)	7.9 (2.3)	*t*(38) = 1.5	.15
FAS fluency (total)	40.2 (12.5)	46.3 (11.4)	*t*(38) = 1.5	.13	46.4 (10.1)	47.8 (17.5)	*t*(37) = 0.32	.75
FAS fluency (perseverations)	2.2 (2.2)	2.0 (2.4)	*t*(38) = 0.55	.89	.95 (1.7)	2.0 (3.9)	*t*(37) = 1.1	.26
Mental control (max. = 40)	25.0 (6.8)	27.4 (5.3)	*t*(36) = 1.2	.23	25.6 (4.3)	25.7 (5.9)	*t*(37) = 0.17	.98
CVLT delay (max. = 16)	10.2 (3.9)	11.6 (2.3)	*t*(33) = 1.3	.22	11.5 (3.3)	11.1 (3.9)	*t*(35) = 0.35	.73
Wisconsin Card Sort, categories (max. = 6)	4.8 (1.6)	4.5 (1.9)	*t*(36) = 0.54	.59	5.3 (1.5)	5.2 (1.7)	*t*(37) = 0.18	.86
Wisconsin Card Sort, errors	4.5 (5.1)	3.6 (6.8)	*t*(36) = 0.49	.63	4.9 (6.2)	5.4 (7.6)	*t*(37) = 0.21	.83
*Note.* There were no significant differences in any measures between the participants who completed Experiment 1 and those who completed Experiment 2 (all *p*s > .10). There were some missing data for cognitive test scores; therefore, degrees of freedom are reported for all *t*-tests. Only the Arithmetic measure showed a significant effect of time of day in Experiment 1. No measures showed a significant effect of time of day in Experiment 2. The Morningness–Eveningness Questionnaire (MEQ) is from Horne and Ostberg (1976), with higher scores representing a morning orientation. The Mini-Mental Status Examination (MMSE) is from Folstein, Folstein, and McHugh (1975). The Shipley Vocabulary test is from Shipley (1986). The arithmetic, digit symbol, and digit span backwards scores are from the corresponding subtests of the Wechsler Adult Intelligence Scale (3rd ed.; Wechsler, 1997a); the digit symbol copy was administered with a 60-s time limit. The FAS fluency task is from Spreen and Benton (1977); scores represent the total number of words generated beginning with the letters *F*, *A*, and *S*, with 60-s time limits for each letter, and the total number of word repetitions (perseverations) generated. The mental control subtest is from the Wechsler Memory Scale (3rd ed.; Wechsler, 1997b). The California Verbal Learning Test (CVLT) is from Delis, Kramer, Kaplan, and Ober (2000) and represents the delayed recall after 30 min. The Wisconsin card sort measures are from Nelson (1976) and represent the number of sorting categories completed and the number of errors made during sorting. max. = maximum value possible on task.								

**Table 2 tbl2:** Mean Recall (Percentages, With Standard Errors in Parentheses) by Group and Condition in Experiment 1

	SP test			IP test		
Group	T	NT	B	T	NT	B
Neutral words						
Younger adults	80.6 (2.8)	66.8 (4.2)	76.9 (4.2)	72.5 (4.1)	61.7 (3.1)	75.3 (3.3)
Older adults, a.m.	65.1 (4.3)	63.3 (4.6)	57.9 (4.0)	66.0 (4.2)	60.8 (4.0)	61.1 (4.7)
Older adults, p.m.	73.0 (3.4)	64.1 (4.1)	54.3 (4.6)	63.4 (3.8)	57.9 (3.8)	56.5 (4.2)
Negative words						
Younger adults	78.5 (3.1)	66.9 (3.9)	76.7 (4.4)	76.2 (3.0)	66.0 (3.0)	76.2 (3.7)
Older adults, a.m.	59.8 (4.1)	59.5 (5.3)	46.4 (5.6)	65.1 (3.9)	59.5 (3.8)	53.1 (6.9)
Older adults, p.m.	66.2 (3.7)	59.9 (2.3)	61.8 (4.7)	59.1 (3.4)	59.1 (3.8)	54.7 (4.7)
*Note.* SP = standard probe; IP = independent probe; T = think; NT = no-think; B = baseline pair.						

**Table 3 tbl3:** Average Endorsement of Potential Strategies Used During Suppression in the Open Instruction Condition in Experiment 1

Statement	YA	OA, a.m	OA, p.m.	*t*(58)
I stared intently at the red word.	2.7^a^	0.9	1.2	5.00
I repeated the red word to myself.	1.2	0.5	0.6	2.35
I used the red word to generate related words or thoughts.	1.3	1.6	1.9	1.30
I used the red word to generate a personal memory.	0.8	0.6	0.5	1.17
I used the red word to generate a sound.	1.2^a^	0.3	0.3	3.95
I stared blankly at the red word and kept my mind clear.	3.1^a^	0.7	1.3	5.71
I refocused my attention on another sensation.	0.7	1.0	0.4	0.16
I refocused my attention on other unrelated thoughts	0.8	1.5	1.0	1.05
I played word games with the red word.	0.6	0.4	0.1	2.06
I refocused my attention on a “distracting” task.	0.8	0.7	0.9	0.15
I diverted my attention away from the cue word.	0.4^a^	2.0	1.4	4.02
I diverted my eyes.	1.0	1.1	0.6	0.58
*Note.* The scale ranged from 0 to 4, with 4 indicating high endorsement. YA = younger adults; OA = older adults.				
^a^ YA score differs significantly from the combined OA a.m. and p.m. groups at a Bonferroni-corrected alpha level of .004.				

**Table 4 tbl4:** Median Split Data of the Top- and Bottom-Performing a.m. and p.m. Older Adults in Experiment 1

Group	Score level	Condition	No-think (%)	Baseline (%)
a.m.	Bottom	SP	45.0	39.0
		IP	56.0	38.8
	Top	SP	75.2	60.4
		IP	60.7	62.9
p.m.	Bottom	SP	55.0	32.5
		IP	54.6	34.7
	Top	SP	67.7	77.6
		IP	71.1	71.3
*Note.* SP = standard probe; IP = independent probe.				

**Table 5 tbl5:** Mean Recall (Percentages, With Standard Errors in Parentheses) by Group and Condition in Experiment 2

	SP test			IP test		
Group	T	NT	B	T	NT	B
Neutral words						
Younger adults	91.0 (2.9)	78.1 (2.9)	86.2 (2.6)	57.8 (3.9)	44.3 (3.2)	57.7 (3.3)
Older adults, a.m.	85.5 (3.1)	67.5 (3.3)	79.7 (3.6)	61.5 (3.5)	49.4 (3.8)	58.3 (3.9)
Older adults, p.m.	76.4 (3.9)	64.5 (3.8)	73.1 (3.6)	59.5 (4.3)	46.4 (3.9)	58.3 (5.3)
Negative words						
Younger adults	87.8 (2.7)	71.9 (2.6)	83.0 (3.7)	69.0 (2.7)	46.3 (4.3)	60.6 (4.0)
Older adults, a.m.	80.9 (3.0)	63.2 (2.9)	78.7 (4.2)	68.0 (2.7)	55.4 (3.3)	67.6 (4.4)
Older adults, p.m.	78.8 (2.8)	64.3 (5.0)	77.5 (4.4)	61.6 (3.9)	42.2 (4.6)	54.5 (5.3)
*Note*. SP = standard probe; IP = independent probe; T = think, NT = no-think; B = baseline pairs.
