# Supplementary material for: Older Adults Can Suppress Unwanted Memories When Given an Appropriate Strategy
Source: Psychol Aging. 2015 Jan 19;30(1):9–25. doi: 10.1037/a0038611 (PMC4360753; doi:10.1037/a0038611)
Supplement: Supplementary file 1 [file z2m004142853so1.docx]

**Supplemental Materials**

**Older Adults can Suppress Unwanted Memories When Given an Appropriate Strategy**

**by B. D. Murray et al., 2014, *Psychology and Aging***

**http://dx.doi.org/10.1037/a0038611**

*Think/No-think Strategy Survey*

Please rate the extent to which you used each of the following strategies in order to keep the response word from coming to mind. ***Please read all of the possible strategies first, before making your ratings.***

***Stared intently at the red word.*** To divert my mind from thinking about the Response word, I focused on the individual letters, or the word’s overall appearance.

Never Rarely Sometimes Often Always

**0 1 2 3 4**

***Repeated the red word to myself.*** To divert my mind from thinking of the Response word, I said the red word to myself again and again to keep my mind occupied.

Never Rarely Sometimes Often Always

**0 1 2 3 4**

***Used the red word to generate related words or thoughts.*** To divert my mind, I used the red word to generate a related word or idea that is associated to the red word.

Never Rarely Sometimes Often Always

**0 1 2 3 4**

***Used the red word to generate a personal memory.*** To divert my mind, I used the red word to generate a memory of an event from my life that was related to the word.

Never Rarely Sometimes Often Always

**0 1 2 3 4**

***Used the red word to generate a sound.*** To divert my mind, I thought of a sound or music related to the hint word.

Never Rarely Sometimes Often Always

**0 1 2 3 4**

***Stared blankly at the red word and kept my mind clear.*** Although I kept looking at the red word, I didn’t think about how it appeared. I just cleared my mind and thought of nothing, as if meditating.

Never Rarely Sometimes Often Always

**0 1 2 3 4**

***Refocused my attention on another sensation.*** When the red word appeared, I kept looking at the word, but I shifted my attention to sounds in the room or outside, or I thought about another sensation (e.g., how comfortable the chair was, etc.)

Never Rarely Sometimes Often Always

**0 1 2 3 4**

***Refocused my attention on other unrelated thoughts.*** When the red word appeared, I kept looking at the word, but I just “checked out” and thought about things unrelated to the experiment (e.g., my to-do list for that day).

Never Rarely Sometimes Often Always

**0 1 2 3 4**

***Played word games with the red word.*** When the red word appeared, I searched for other words that could be made from the letters, counted the number of letters, etc.

Never Rarely Sometimes Often Always

**0 1 2 3 4**

***Refocused my attention on a “distracting” task.*** For instance, you might have counted backward in your head or done some other task to keep yourself occupied.

Never Rarely Sometimes Often Always

**0 1 2 3 4**

***Diverted my attention away from the cue word.*** Although I kept my eyes on the screen, I shifted my attention covertly to a different location (e.g., above the word, in the corner of the screen, etc.) so that I could avoid looking at the red word.

Never Rarely Sometimes Often Always

**0 1 2 3 4**

***Diverted my eyes.*** When the red word appeared, I moved my eyes away from the word and the screen so that I did not look at it.

Never Rarely Sometimes Often Always

**0 1 2 3 4**

*Note:* “Hint word” was the terminology used with the participants rather than “cue word”.

Supplementary Results

Because interactions with emotion were not found in any analyses, in the main body of the paper, we do not present separate analyses for memory suppression for the negative pairs and the neutral pairs. In this supplementary results section, we analyze the results for the negative pairs separately from the results for the neutral pairs.

**Experiment 1: Suppression for Negative Items.**

An ANOVA with task (SP, IP) and memory control condition (Think, No-think, Baseline) as within-subject factors and group (Young, Older AM, Older PM) as a between-subject factor revealed a main effect of memory control, *F*(2,56)=5.26, *p*<.01, partial eta-squared = .16, as well as an interaction between memory control and group, *F*(4,114)=2.74, *p*<.05, partial eta-squared = .09. No other main effects or interactions reached significance (all *F*<1.5).

To clarify the basis for the memory control x age interaction, each group was analyzed separately, in an ANOVA with task (SP, IP) and memory control condition (Think, No-think, Baseline) as within-subject factors. In younger adults, this ANOVA revealed a significant effect of memory control condition (T=B>NT, *F*(2,18)=6.95, *p*<.01, partial eta-squared = .44) but no effect of task and no interaction (*F*s<1). For the older adults tested in the morning, the ANOVA also revealed only a main effect of memory control condition (*F*(2,18)=3.72, *p*<.05, partial eta-squared = .29), but in this group it was due to a different pattern, whereby No-think as well as Think words were remembered above Baseline (T=NT>B); in other words, there was *facilitation* for the No-think words. In the older adults tested in the evening, there were no significant effects or interactions.

These results confirm that the pattern described in the manuscript for Experiment 1 holds when only negative items are analyzed: Younger adults show memory suppression even when they are given no particular strategies to achieve suppression, whereas older adults do not.

**Experiment 1: Suppression for Neutral Items.**

An ANOVA with task (SP, IP) and memory control condition (Think, No-think, Baseline) as within-subject factors and group (Young, Older AM, Older PM) as a between-subject factor revealed a main effect of memory control, *F*(2,56)=7.12, *p*<.005, partial eta-squared = .20, as well as an interaction between memory control and group, *F*(4,114)=2.9, *p*<.05, partial eta-squared = .09. No other main effects or interactions reached significance (all *F*<2.5).

To clarify the basis for the memory control x age interaction, each group was analyzed separately, in an ANOVA with task (SP, IP) and memory control condition (Think, No-think, Baseline) as within-subject factors. In younger adults, this ANOVA revealed a significant effect of memory control condition (T=B>NT, *F*(2,18)=8.68, *p*<.005, partial eta-squared = .49) but no effect of task and no interaction (*F*s<1.7). For the older adults tested in the morning, the ANOVA revealed no significant effects or interactions (*F*s < 1). For the older adults tested in the afternoon, there was a significant effect of test (SP>IP, *F*(1,19)=4.92,*p*<.05, partial eta-squared = .21). There was a significant effect of memory control condition (*F*(2,18)=4.47, *p*<.05, partial eta-squared = .33), with Think words remembered better than either No-Think or Baseline words (T>B=NT). There was no evidence of memory suppression, and recall of No-Think words was numerically higher (.61) than Baseline words (.55).

These results confirm that the pattern described in the manuscript for Experiment 1 holds when only neutral items are analyzed. When provided with no particular strategy, younger adults show memory suppression but older adults do not.

**Experiment 2: Suppression for Negative Items.**

An ANOVA with task (SP, IP) and memory control condition (Think, No-think, Baseline) as within-subject factors and group (Young, Older AM, Older PM) as a between-subject factor revealed a main effect of task (SP>IP, *F* (1,57) =54.04, *p* <.001, partial eta-squared =.47) and a main effect of memory control, (T>B>NT, *F*(2,56)=38.15, *p*<.001, partial eta-squared = .58). No other effects were significant, *F*s<1.1.

Although interactions with group were not significant, for completeness we present the results of ANOVAs conducted separately in each group. For all groups, there was a main effect of task, with more items remembered in the SP than IP task (younger adults: *F*(1,19)=34.28, *p* <.001, partial eta-squared = .64; morning older adults: *F*(1,19)=5.81, *p* <.05, partial eta-squared = .23; afternoon older adults: *F*(1,19)=22.28, *p* <.001, partial eta-squared = .54). For all groups there also was a main effect of memory control condition (younger adults: *F*(2,18)=20.19, *p* <.001, partial eta-squared = .69; morning older adults: *F*(2,18)=16.96, *p* <.001, partial eta-squared = .65; afternoon older adults: *F*(2,18)=7.56, *p* <.01, partial eta-squared = .46). Critically, in each age group, No-think words were remembered less well than were words in either the Think condition or in the Baseline condition. These comparisons were significant for all comparisons in the SP and IP tasks (all *t*(19)>2.44, *p*<.05) with one exception: For afternoon older adults, the difference between No-think and Baseline items was only marginally significant on the Independent Probe task, *t*(19)=1.72, *p*=.10.

These results confirm that the pattern described in the manuscript for Experiment 2 holds when only negative items are analyzed: When given directed instructions, both younger and older adults show memory suppression for negative items on both the Independent Probe and Same Probe tasks.

**Experiment 2: Suppression for Neutral Items.**

An ANOVA with task (SP, IP) and memory control condition (Think, No-think, Baseline) as within-subject factors and group (Young, Older AM, Older PM) as a between-subject factor revealed a main effect of task (SP>IP, *F* (1,57) =91.26, *p* <.001, partial eta-squared =.62), an interaction between task and group (*F* (2,57)=3.5, *p*<.05, partial eta-squared = .11), as well as a main effect of memory control (T>B>NT, *F*(2,56)=31.29, *p*<.001, partial eta-squared = .53). No other effects or interactions were significant, *F*s<1.

For all groups, there was a main effect of task (SP > IP; younger adults: *F*(1,19)=67.05, *p* <.001, partial eta-squared = .78; morning older adults: *F*(1,19)=31.3, *p* <.001, partial eta-squared = .62; afternoon older adults: *F*(1,19)=11.63, *p* <.001, partial eta-squared = .38). For all groups there also was a main effect of memory control condition (younger adults: *F*(2,18)=21.75, *p* <.001, partial eta-squared = .71; morning older adults: *F*(2,18)=11.04, *p* <.005, partial eta-squared = .55; afternoon older adults: *F*(2,18)=5.96, *p* <.05, partial eta-squared = .40). No group showed a significant interaction between test and memory control condition (*F*s < 1). Critically, in each age group, No-think words were remembered less well than were words in either the Think condition or in the Baseline condition. These comparisons were significant for all comparisons in the SP and IP tasks (all *t*(19)>2.14, *p*<.05) with two exceptions: For morning and afternoon older adults, the difference between No-think and Baseline items was of marginal significance on the Independent Probe test (morning: *t*(19)=1.72, *p*=.059; afternoon: *t*(19)=2.07, *p*=.052).

These results confirm that the pattern described in the manuscript for Experiment 2 holds when only neutral items are analyzed. Younger and older adults show memory suppression for neutral items on both the Independent Probe and Same Probe tasks when they are given directed instructions.
